# Inhibition of USP1 enhances anticancer drugs-induced cancer cell death through downregulation of survivin and miR-216a-5p-mediated upregulation of DR5

**DOI:** 10.1038/s41419-022-05271-0

**Published:** 2022-09-24

**Authors:** Seon Min Woo, Seok Kim, Seung Un Seo, Shin Kim, Jong-Wook Park, Gyeonghwa Kim, Yu-Ra Choi, Keun Hur, Taeg Kyu Kwon

**Affiliations:** 1 Department of Immunology, School of Medicine, Keimyung University, Daegu, 42601 South Korea; 2 Department of Biochemistry and Cell Biology, School of Medicine, Kyungpook National University, Daegu, 41944 South Korea; 3 Center for Forensic Pharmaceutical Science, Keimyung University, Daegu, 42601 South Korea

**Keywords:** Apoptosis, Targeted therapies

## Abstract

Ubiquitin-specific protease 1 (USP1) is a deubiquitinase involved in DNA damage repair by modulating the ubiquitination of major regulators, such as PCNA and FANCD2. Because USP1 is highly expressed in many cancers, dysregulation of USP1 contributes to cancer therapy. However, the role of USP1 and the mechanisms underlying chemotherapy remain unclear. In this study, we found high USP1 expression in tumor tissues and that it correlated with poor prognosis in RCC. Mechanistically, USP1 enhanced survivin stabilization by removing ubiquitin. Pharmacological inhibitors (ML23 and pimozide) and siRNA targeting USP1 induced downregulation of survivin expression. In addition, ML323 upregulated DR5 expression by decreasing miR-216a-5p expression at the post-transcriptional level, and miR-216a-5p mimics suppressed the upregulation of DR5 by ML323. Inhibition of USP1 sensitized cancer cells. Overexpression of survivin or knockdown of DR5 markedly prevented the co-treatment with ML323 and TRAIL-induced apoptosis. These results of in vitro were proved in a mouse xenograft model, in which combined treatment significantly reduced tumor size and induced survivin downregulation and DR5 upregulation. Furthermore, USP1 and survivin protein expression showed a positive correlation, whereas miR-216a-5p and DR5 were inversely correlated in RCC tumor tissues. Taken together, our results suggest two target substrates of USP1 and demonstrate the involvement of survivin and DR5 in USP1-targeted chemotherapy.

## Introduction

Post-translational modification (PTM) is a covalent process that regulates protein biosynthesis through phosphorylation, ubiquitination, glycosylation, and SUMOylation and is critical in many biological signaling events through the alteration of proteolytic cleavage-dependent protein properties [1]. Ubiquitination, a PTM, is involved in the degradation of proteins through the ubiquitin-proteasome system and plays a significant role in numerous biological processes, including DNA repair, trafficking events, and signal transduction [2]. Ubiquitination is catalyzed by an enzyme complex that contains E1 activating, E2 conjugating, and E3 ligase enzymes. In contrast, it can be inhibited by deubiquitinases (DUBs), which remove ubiquitin from target proteins [3, 4]. In humans, about 100 DUBs are categorized into six classes according to their catalytic domain: five cysteine proteases [ubiquitin-specific proteases (USPs), ubiquitin C-terminal hydrolases (UCHs), ovarian tumor proteases (OTUs), Machado-Joseph disease proteases (MJDs), and MIU-containing novel DUB family (MINDY) protease] and one metalloprotease [Jab1/MPN/MOV34 metalloenzymes (JAMMs)] [5, 6]. Since dysregulation of ubiquitination by DUBs causes diverse diseases, especially cancers, regulation of DUBs may be a strategic target in cancer therapies.

USP1 is a well-characterized member of the USP family and possesses a conserved USP domain containing three catalytic residues: Cys90, His593, and Asp751 [7, 8]. It acts as a key regulator of the DNA repair response, including the Fanconi anemia (FA) pathway and DNA translesion synthesis (TLS), by forming a complex with USP1 associated factor 1 (UAF1) [9]. USP1 impedes mono-ubiquitinated FANCD2, which stops the DNA damage response by preventing the FA pathway [10]. USP1 detaches mono-ubiquitin from PCNA, which is a DNA replication component, and interrupts the recruitment of TLS polymerases, thereby extinguishing DNA double-strand breaks in DNA translesion synthesis [11].

Overexpression of USP1 is commonly observed in osteosarcoma and colorectal, non-small cell lung, and gastric cancers [8, 12–14], and the blockade of USP1 causes apoptosis in many cancers [15–17]. Several USP1 inhibitors sensitizes cancer cell to platinum-, DNA-damaging-, and radiation-induced death [13, 18–20]. These reports suggest the significance of USP1 as a hopeful therapeutic target in cancer chemotherapy. Above all, ML323 selectively and allosterically inhibits formation of USP1/UAF1 complex and potentiates mono-ubiquitination of both FANCD2 and PCNA [9, 21]. Moreover, ML323 increases sensitivity to doxorubicin in colorectal cancer and overcomes resistance to cisplatin in non-small cell lung cancer [13, 20]. However, the effects and detailed molecular mechanisms of ML323 on cancer cell death remain unclear.

In this study, we identified the target substrate of USP1 and investigated its molecular mechanisms and USP1 inhibition as a sensitizer for chemotherapy.

## Materials and methods

### Patient specimens

Forty renal clear carcinoma specimens were collected from the Keimyung University Dongsan Hospital Biobank (IRB-2019-11-040).

### Survival rate of patients with renal clear carcinoma (RCC)

The overall survival data of approximately 527 patients with RCC were obtained using ESurv (Easy, Effective, and Excellent Survival analysis tool, https://easysurv.net) based on The Cancer Genome Atlas (TCGA) [22]. The overall survival data of approximately 124 patients with RCC were obtained using Survival Genie (https://bbisr.shinyapps.winship.emory.edu/SurvivalGenie/) based on Therapeutically Applicable Research to Generate Effective Treatments (TARGET) [23].

### Cells and materials

Caki-1, ACHN, A549, HCT116, and SK-Hep1 cells were obtained from the American Type Culture Collection (Manassas, VA, USA) and cultured in Dulbecco’s modified Eagle’s medium or Roswell Park Memorial Institute 1640 (Welgene, Gyeongsan, Korea) containing 10% fetal bovine serum (FBS) (Welgene), 5% penicillin/streptomycin (Welgene), and 100 μg/mL gentamicin (Thermo Fisher Scientific, Waltham, MA, USA). ML323 (18010) was obtained from Cayman Chemical Co. (Ann Arbor, MI, USA). Cycloheximide (01810), actinomycin D (A1410), cisplatin (P4394), carboplatin (C2538), doxorubicin (D1515), and etoposide (E1383) were obtained from Sigma-Aldrich (St. Louis, MO, USA), and MG132 (474790) was purchased from Calbiochem (San Diego, CA, USA), and. Human recombinant TRAIL (375-TL) and z-VAD-fmk (FMK001) were obtained from R&D Systems (Minneapolis, MN, USA). Anti-Fas (05-201) was obtained from Merck Millipore (Darmstadt, Germany). The primary antibodies were supplied as follows: anti-USP1 (A301-698, 1:700) from Bethyl Laboratories (Waltham, MA, USA); anti-Bcl-2 (ab196495, 1:700) and anti-DR4 (ab8414, 1:1000) from Abcam (Waltham, MA, USA); anti-Mcl-1 (sc-12756, 1:700) and anti-cIAP2 (sc-7944, 1:1000) from Santa Cruz Biotechnology (Santa Cruz, CA, USA); anti-Bax (554104, 1:700), anti-Bim (AB17003, 1:700), and anti-XIAP (610762, 1:1000) from Biosciences (San Jose, CA, USA); anti-survivin (AF886, 1:700) from R&D System; anti-Bcl-xL (#2764, 1:1000), anti-DR5 (#8074, 1:700), and anti-PARP (#9542, 1:700) from Cell Signaling Technology (Beverly, MA, USA); anti-c-FLIP (ALX-804-961-0100, 1:700) from Enzo Life Sciences (San Diego, CA, USA).

### Transfection

Green fluorescent protein (control) and USP1 siRNA were obtained from Bioneer (Daejeon, Korea). The siRNAs sequence of human USP1 are as follows: siRNA-1: 5′-GCAUAGAGAUGGACAGUAU-3′; siRNA-2: 5′-GGUUGCUAGUACAGCGUUU-3′. siRNA or plasmid transfection were conducted using Lipofectamine RNAiMAX (Thermo Fisher Scientific) or Lipofectamine p-MAX (AptaBio, Yongin, Korea), respectively.

### Analysis of protein and mRNA expression

To examine the alteration of protein expression, western blotting progressed as described previously [24–26]. Obtained proteins using RIPA lysis buffer were separated using sodium dodecyl sulfate–polyacrylamide gel electrophoresis, followed by transfer to Immobilon-P membrane (GE Healthcare Life Science, Pittsburgh, PO, USA). The membranes were incubated with primary antibodies for overnight, and then the membranes were incubated with secondary antibodies for 2 h. Finally, membranes were visualized using an enhanced chemiluminescence kit (Merck Millipore). Full-length original western blots in this article are shown in Supplemental Material. For the analysis of mRNA expression, RT-PCR and quantitative PCR (qPCR) used as described previously [27]. The following primers were used to amplify the target genes for RT-PCR and qPCR, respectively: survivin (forward) 5′-GGACCACCGCATCTCTACAT-3′ and (reverse) 5′-GCACTTTCTTCGCAGTTTCC-3′, DR5 (forward) 5′-AAGACCCTTGTGCTCGTTGT-3′ and (reverse) 5′-GACACATTCGATGTCA CTCCA-3′, and actin (forward) 5′-GGCATCGTCACCAACTGGGAC-3′ and (reverse) 5′-CG ATTTCCCGCTCGGCCGTGG-3′ for RT-PCR; survivin (forward) 5′-TTCTCAAGGACCA CCGCATC-3′ and (reverse) 5′-GTTTCCTTTGCATGGGGTCG-3′, DR5 (forward) 5′-AGACCCTTGTGCTCGTTGTC-3′ and (reverse) 5′-TTGTTGGGTGATCAGAGCAG-3′, and actin (forward) 5′-CTACAATGAGCTGCGTGTG-3′ and (reverse) 5′-TGGGGTGTTGAA GGTCTC-3′ for qPCR.

### Immunoprecipitation and ubiquitination assays

Cells were harvested and sonicated in RIPA lysis buffer containing 10 mM N-ethylmaleimide (Sigma-Aldrich) and 1 mM phenylmethylsulfonyl fluoride (Sigma-Aldrich) on ice. Experimental methods were described in a previous study [28]. In brief, the cell lysates were incubated overnight with anti-survivin antibody (Cell Signaling Technology) and subsequently with protein PLUS-Agarose (Santa Cruz Biotechnology) for 2 h at 4 °C. Ubiquitination assay was performed using horseradish peroxidase-conjugated anti-Ub (Enzo Life Sciences) under denaturation conditions.

### Cell surface levels of DR5

For cell surface expression analysis of DR5, the cells were incubated with DR5-phycoerythrin (Abcam) in phosphate-buffered saline (PBS) containing 10% FCS and 1% sodium azide. The samples were processed and analyzed by flow cytometry (BD Biosciences, San Jose, CA, USA).

### Measurement of luciferase activity

Cells were transfected with DR5 (−605)-, DR5 (SacI)-, DR5 3′-UTR WT-, DR5 3′-UTR miR-216a-5p mutant-, or pGL2-miR-216-luciferease promoter. Measurement of luciferase assay was performed using the luciferase substrate luciferin (Promega, Madison, WI, USA).

### microRNAs expression analysis

MicroRNAs were extracted from tissue specimens using the QIAzol lysis reagent (Qiagen, CA, USA) according to the manufacturer’s instructions. The quantity and quality of total miRNAs were measured using a NanoPhotometer N60 (Implen NanoPhotometer, Westlake Village, CA, USA). The relative expression levels of miRNAs in RCC tissue samples were determined using TaqMan microRNA assays (Applied Biosystems, Foster City, CA, USA). The qRT-PCR data for miRNA were normalized to RNU6B, as an endogenous control, using the 2−ΔΔCt method.

### Detection of apoptosis

To verify apoptosis, we used diverse experimental procedures. For sub-G1 analysis, fixed cells by 100% ethanol were incubated with RNase for 30 min at 37 °C, stained with propidium iodide (Sigma-Aldrich), and then measured by flow cytometry (BD Biosciences). For measurement of caspase-3 (DEVDase) activation, cell lysates were incubated with anacetyl-Asp-Glu-Val-Asp p-nitroanilide (Ac-DEVD-pNA), and their activity were measured by a spectrophotometer. For nuclear condensation, cells were stained with 4′, 6′-diamidino-2-phenylindole solution (Roche, Mannheim, Germany), and fluorescence images were analyzed using fluorescence microscopy (Carl Zeiss, Jena, Germany).

### Mouse xenograft model

Male BALB nude mice (Bagg and albino) were purchased from JA Bio, Inc. (Suwon, Korea). HCT116 cells were subcutaneously injected into the flank of each mouse. Mice were randomized into two groups (six mice per group) for treatment with either vehicle [2% dimethyl sulfoxide (DMSO)/PBS] or 10 mg/kg ML323 three times a week, an intraperitoneal injection. Tumor size was calculated using the formula [(length × width^2^)/2]. To prove apoptosis in vivo sample, we performed Terminal deoxynucleotidyl transferase (TdT) dUTP Nick-End Labeling (TUNEL) assay using the ApopTag Fluorescein in situ Apoptosis Detection Kit (Merck Millipore). Animal experiments were approved by Keimyung University Ethics Committee (KM-2020-03R2).

### Statistical analysis

The data were analyzed using one-way ANOVA and post hoc comparisons (Student-Newman-Keuls) using the Statistical Package for Social Sciences software (version 22.0; SPSS Inc., Chicago, IL, USA). Significant differences in miRNA expression were calculated using paired Student’s t-test and correlation analysis.

## Results

### USP1 is highly expressed in the tissues of patients with human renal clear carcinoma (RCC)

**Fig. 1:**
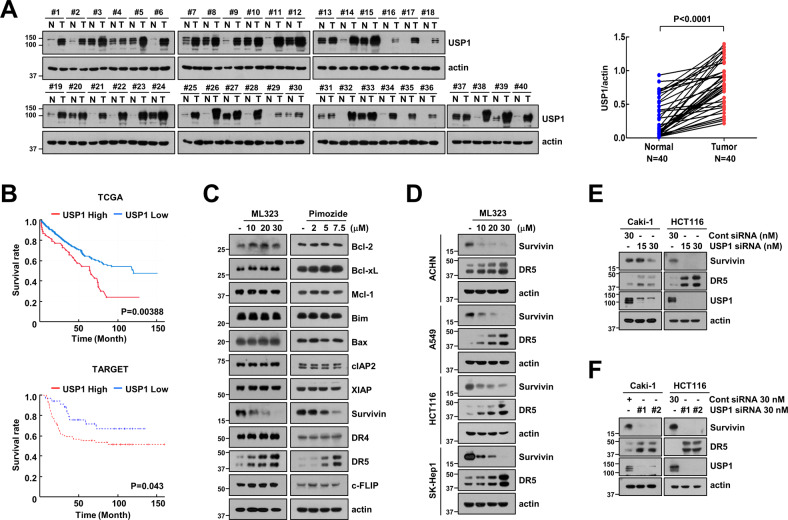
USP1 is upregulated in human renal clear carcinoma (RCC) tissues. **A** Examination of USP1 protein expression in 40 paired primary RCC tissues and corresponding normal adjacent tissues. **B** Analysis of prognostic significance of USP1 in patients with RCC based on The Cancer Genome Atlas (TCGA, *n* = 527, upper) and Therapeutically Applicable Research to Generate Effective Treatments (TARGET, *n* = 124, lower). Investigation of apoptosis-related protein expression in cancer cell lines treated to 10–30 μM ML323 (**C**) or 2–7.5 μM pimozide (**D**) for 24 h. **E, F** The cancer cells were transfected with control siRNA or USP1 siRNA for 24 h. Protein expression was measured using western blotting (**A**, **C**–**F**).

To validate the clinical relevance of USP1 expression in patients with RCC (*n* = 40), we performed immunoblotting to analyze the expression levels of USP1 in a panel of non-tumor and tumor tissues. The expression levels of USP1 was higher in tumor tissues than in non-tumor tissues (Fig. 1A). To determine the overall survival rate, prognostic relevance of USP1 was analyzed by correlating baseline USP1 expression with the overall survival of RCC. Patients with high-expression levels of USP1 had shorter overall survival time than those with low expression levels in TCGA dataset (*P* = 0.00388) and TARGET dataset (*P* = 0.043) (Fig. 1B). We performed the univariate and multivariate analyses in 418 RCC patients. Univariate analysis showed that age (*p* = 0.006), stage (*p* < 0.001), and smoking (*p* = 0.002) correlate with overall survival of RCC patients. In multivariate analysis, smoking and stage (*p* < 0.001) were related to RCC patient survival (Supplementary Table. S1). To investigate whether the differential expression pattern of apoptosis-related proteins caused USP1 inhibition, we explored the changes in apoptosis-associated proteins induced by USP1 specific inhibitors (ML323 and pimozide) in Caki-1 cells. Significant differences in the expression of these two proteins were detected after treatment with USP1 inhibitors. DR5 expression levels were increased after 24 h in the two USP1 inhibitor treatments, whereas survivin expression was downregulated. However, the expression of other proteins (Bcl-2 and IAP family proteins, DR4, and c-FLIP) was not changed by the two USP1 inhibitors (Fig. 1C). Next, we investigated the effect of ML323 on diverse cancer cell lines (renal carcinoma, ACHN; lung carcinoma, A549; colon carcinoma, HCT116; and hepatocellular carcinoma, SK-Hep1). ML323 induced a similar expression pattern in all tested cells (Fig. 1D). To rule out the off-target effects of USP1 inhibitors, we used specific siRNA for USP1. USP1 knockdown induced survivin downregulation and DR5 upregulation (Fig. 1E, F). Therefore, these data suggested that the pharmacological and genetic inhibition of USP1 induce changes in DR5 and survivin protein expression.

### USP1 regulates survivin stability

**Fig. 2:**
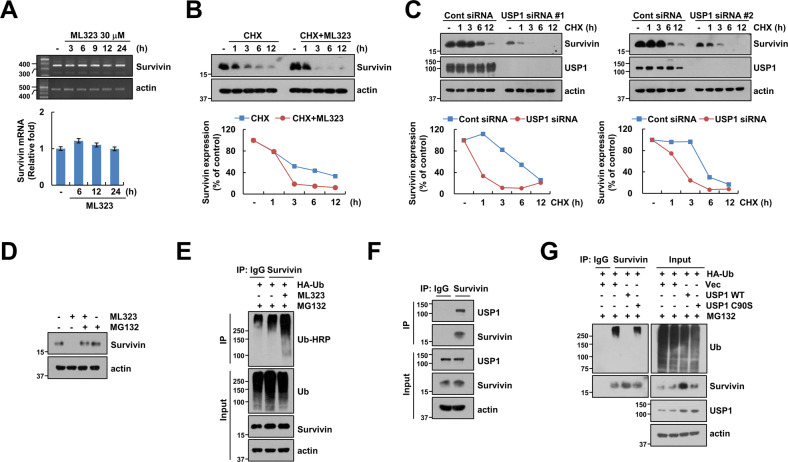
USP1 directly interacts and induces deubiquitination of survivin. **A** Caki-1 cells were treated with 30 μM ML323 for the indicated time periods, and survivin mRNA expression were analyzed using RT-PCR and qPCR. **B** Caki-1 cells were pretreated with 20 μg/mL cycloheximide (CHX) for 30 min and then treated with 30 μM ML323 for the indicated time periods. **C** Caki-1 cells were transfected with control siRNA or USP1 siRNA followed by treatment with 20 μg/mL CHX for the indicated time periods. **D** Caki-1 cells were pretreated with 0.25 μM MG132 for 30 min and then treated with 30 μM ML323 for 24 h. **E** Caki-1 cells were transfected with the HA-ubiquitin (HA-Ub) followed by combinations of 0.25 μM MG132 and 30 μM ML323 for 12 h. Ubiquitination assay of survivin was performed using an anti-survivin antibody. **F** Investigation of interaction between USP1 and survivin for IP assay using an anti-survivin antibody. **G** Caki-1 cells were transfected with vector, USP1 WT or USP1 C90S and HA-ubiquitin followed by 0.25 μM MG132 for 12 h. Protein expression was measured using western blotting (**B**–**G**). The band intensity of survivin protein was quantified by using ImageJ.

ML323 downregulated the protein level of survivin in a time-dependent manner, whereas the mRNA expression level of survivin mRNA was not downregulated by ML323 treatment (Fig. 2A). We examined whether USP1 inhibition modulates survivin protein stability. Caki-1 cells were treated with or without a pharmacological USP1 inhibitor (ML323) and USP1 siRNA in the presence of cycloheximide (CHX) for various time points. Combined treatment with CHX and ML323 or USP1 siRNA significantly decreased survivin stability compared to CHX alone (Fig. 2B, C). In addition, pretreatment with MG132, a proteasome inhibitor, prevented the ML323-induced survivin downregulation (Fig. 2D). Further, we investigated ML323-mediated survivin ubiquitination and found that ML323 markedly increases the polyubiquitination of survivin (Fig. 2E). To further identify which USP1 directly modulates the ubiquitination of survivin, the interaction between USP1 and survivin was assessed using an immunoprecipitation assay. USP1 directly bound to survivin (Fig. 2F). Furthermore, USP1 dramatically inhibited the ubiquitination of survivin, while the catalytic mutant, C91S, of USP1 increased the ubiquitination of survivin (Fig. 2G). These data indicated that USP1 inhibition decreases survivin stabilization via the induction of survivin ubiquitination.

### ML323 upregulates DR5 expression at the post-transcriptional level

**Fig. 3:**
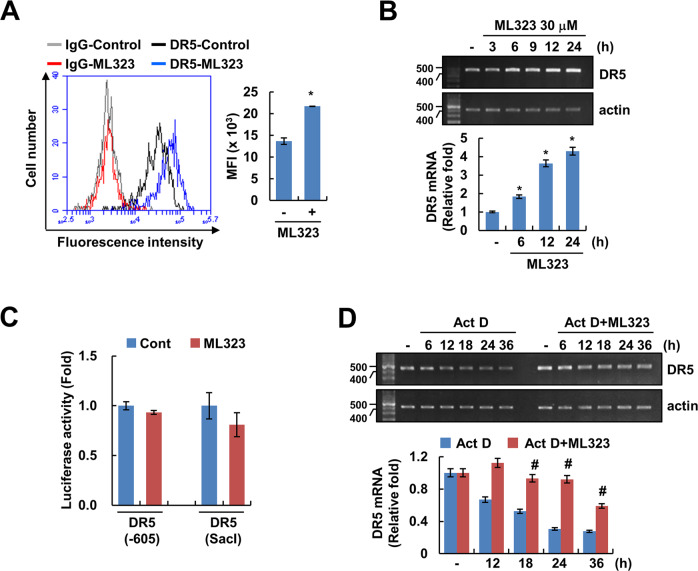
ML323 upregulates DR5 expression at the post-transcription level. **A** Investigation of DR5 surface expression in Caki-1 cells treated to 30 μM ML323 for 12 h. **B** Investigation of DR5 mRNA expression using RT-PCR and qPCR in Caki-1 cells treated to 30 μM ML323 for the indicated time periods. **C** Caki-1 cells were transiently transfected with DR5 (−605) or DR5 (SacI) promoter plasmids, treated with 30 μM ML323 for 12 h, and analyzed by luciferase activity assay. **D** Caki-1 cells were pretreated with 2 μg/mL actinomycin D (Act D) for 30 min and then treated with 30 μM ML323 for the indicated time periods. DR5 mRNA expression were analyzed using RT-PCR and qPCR. Values in the graphs (**A**–**D**) represent the mean ± SD of three independent experiments. *P < 0.01 compared to the control. #*P* < 0.01 compared to Act D.

Expression of DR5 on the cell surface is essential for the increase of DR5 protein expression and death receptor (DR)-mediated apoptosis [29]. ML323 induced upregulation of DR5 surface expression (Fig. 3A). Interestingly, ML323 upregulated DR5 protein but not DR4 (Fig. 1C). Therefore, we investigated the possibility of ML323-induced DR5 upregulation at the transcriptional level. Result of mRNA expression followed by PCR assays indicated that ML323 induces upregulation of DR5 mRNA (Fig. 3B). Caki-1 cells were transfected with the DR5 promoter (DR5/−605 or DR5/SacI) and were treated with or without ML323. ML323 did not induce DR5 promoter activity (Fig. 3C). The regulation of DR5 mRNA stability by ML323 was investigated using actinomycin D, which is inhibit de novo transcription. RT-PCR and qPCR showed that ML323 markedly enhances DR5 mRNA stabilization compared to actinomycin D (Fig. 3D). These data demonstrated that ML323 upregulates DR5 through augmentation of DR5 mRNA stability.

### Suppression of miR-216a-5p is critical for upregulation of DR5 mRNA in ML323-treated cells

**Fig. 4:**
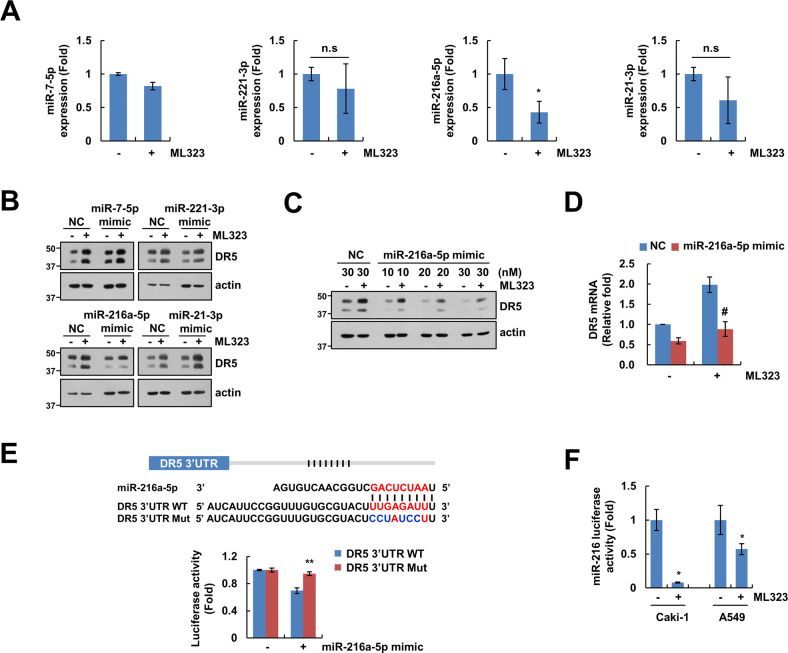
Decrease of miR-216a-5p is involved in ML323-mediated DR5 mRNA upregulation. **A** Investigation of microRNAs in Caki-1 cells treated to 30 μM ML323 for 24 h. **B** Caki-1 cells were transfected with miR-negative control (NC), miR-7-5p mimic, miR-221-3p mimic, miR-216a-5p mimic, or miR-21-3p mimic followed by 30 μM ML323 for 24 h. **C, D** Caki-1 cells were transfected with miR-NC or miR-216a-5p mimic followed by 30 μM ML323 for 24 h. DR5 protein and mRNA expression were analyzed using western blotting (**C**) and qPCR (**D**). **E** Caki-1 cells were transfected with luciferase reporter gene for DR5 3′UTR WT or mutant followed by 30 μM ML323 for 24 h and analyzed using luciferase activity assay. **F** Caki-1 and A549 cells were transfected with miR-216 promoter plasmid followed by 30 μM ML323 for 12 h. Protein expression was measured by western blotting (**B**, **C**). Values in the graphs (**A**, **D**–**F**) represent the mean ± SD of three independent experiments. *P < 0.01 compared to the control. #*P* < 0.01 compared to ML323 treated miR-NC-transfected group. **P < 0.01 compared to miR-216a-5 mimic-treated DR5 3′UTR WT.

To understand mechanisms for USP1 inhibition-mediated DR5 mRNA upregulation, we investigated the relevance of microRNAs (miRNAs). We used TargetScan and GeneCards programs to explore the target miRNAs for DR5 post-transcriptional regulation. We identified that the DR5 3′-UTR region contained four target sites of miRNAs: miR-7-5p, miR-221-3p, miR-216a-5p, and miR-21-3p, and explored the expression of these miRNAs in ML323-treated cells using qPCR. Only miR-216a-5p expression was markedly lower in cells treated with ML323 (Fig. 4A). To further assess which miRNAs were associated with the upregulation of DR5 mRNA by USP1 inhibition, we used mimics for each miRNA. When cells were treated ML323 after transfection of mimics, mimic of miR-216a-5p solely inhibited ML323-mediated DR5 protein upregulation (Fig. 4B, C). Additionally, the miR-216a-5p mimic suppressed ML323-increased DR5 mRNA expression (Fig. 4D). To confirm the relation between DR5 and miR-216a-5p, we generated luciferase reporters containing mutated (Mut) of the miR-216a-5p binding site in the 3′-UTR of DR5 (Fig. 4E). These luciferase plasmids together with the miR-216a-5p mimic showed the decrease of luciferase activity in the DR5 3′-UTR WT but not the Mut (Fig. 4E). Furthermore, ML323 treatment decreased the miR-216a-5p luciferase activity in Caki-1 and A549 cells (Fig. 4F). These results indicated that miR-216a-5p clearly binds to the 3′-UTR of DR5 mRNA and impairs DR5 mRNA expression. Taken together, these results suggested that USP1 inhibition increases DR5 mRNA through downregulation of miR-216a-5p expression.

### ML323 sensitizes anticancer drugs-mediated apoptosis

**Fig. 5:**
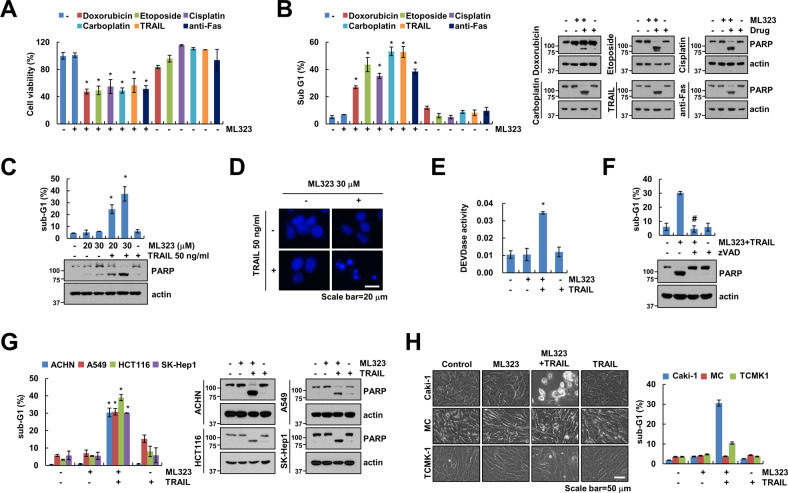
ML323 increases sensitivity to anticancer drugs-mediated apoptosis in cancer cells. **A**, **B** Caki-1 cells were treated with a combination of 1 μM doxorubicin, 3 μg/mL etoposide, 30 μM cisplatin, 200 nM carboplatin, 50 ng/ml TRAIL, and 500 ng/mL anti-Fas in the presence or absence of 30 μM ML323 for 24 h. Cell viability was analyzed using the 2,3-bis(2-methoxy-4-nitro-5-sulfophenyl)-2H-tetrazolium-5-carboxyanilide (XTT) assay kit (**A**). **C**–**E** Caki-1 cells were treated with 30 μM ML323, 50 ng/mL TRAIL or combination for 24 h. Nuclear condensation was determined using DAPI staining. scale bar: 20 μm (**D**). DEVDase (caspase-3) colorimetric assay using DEVDase substrate (**E**). **F** Caki-1 cells were pretreated with 20 μM zVAD for 30 min and then treated with combination of 30 μM ML323 and 50 ng/mL TRAIL for 24 h. **G, H** Cancer (**G**) or normal cell (**H**) lines were treated with 30 μM ML323, 50 ng/mL TRAIL or combination for 24 h. Cell morphology was assessed using a microscope. scale bar: 50 μm (**H**). The sub-G1 population and protein expression were determined using flow cytometry and western blotting, respectively (**B**, **C**, **F**–**H**). Values in the graphs (**A**–**C**, **E**–**H**) represent the mean ± SD of three independent experiments. *P < 0.01 compared to the control. #*P* < 0.01 compared to combination of ML323 and TRAIL.

Next, we investigated whether ML323 sensitizes cells to anticancer drugs. Combined treatment with a sub-lethal dosage of ML323 and anticancer drugs significantly reduced cell viability and induced apoptosis, but not ML323 alone (Fig. 5A, B and Supplementary Fig. S2A). Moreover, combinations of ML323 and anticancer drugs increased phosphorylation of γH2AX expression, a biomarker of DNA double-strand breaks (Supplementary Fig. S1). ML323 induced an increase in the expression of DR5, a TRAIL receptor (Fig. 3A). Therefore, we focused on the ML323-mediated sensitization of TRAIL-induced apoptosis. ML323 alone (20 and 30 μM) or TRAIL alone (50 ng/mL) did not induce apoptosis. However, combinations of ML323 and TRAIL markedly increased the accumulation of the sub-G1 population and cleavage of poly (ADP-ribose) polymerase (PARP) (Fig. 5C and Supplementary Fig. S2B). Also, co-treatment induced nuclear condensation and fragmented DNA, but this was not observed in individual treatment (Fig. 5D). The involvement of caspase activation in combination with ML323 and TRAIL was further examined, which revealed that the combined treatment significantly activated caspase-3 (DEVDase) (Fig. 5E). In addition, z-VAD-fmk, a pan-caspase inhibitor, critically inhibited the combined treatment-induced apoptosis and PARP cleavage (Fig. 5F and Supplementary Fig. S2C). Next, we investigated the sensitizing effect of ML323 in other cancer cells (renal carcinoma ACHN, lung carcinoma A549, colon carcinoma HCT116, and hepatocellular carcinoma SK-Hep1). In all tested cells, the combinations of ML323 and TRAIL increased apoptosis and PARP cleavage (Fig. 5G and Supplementary Fig. S2D), but normal human mesangial cells (MC) and normal mouse kidney cells (TCMK-1) did not cause the increase of sub-G1 (Fig. 5H and Supplementary Fig. S2E). Taken together, these results suggested that ML323 selectively sensitizes cancer cells to TRAIL-mediated apoptosis.

### Depletion of USP1 enhances TRAIL-induced apoptosis through survivin downregulation and DR5 upregulation

**Fig. 6:**
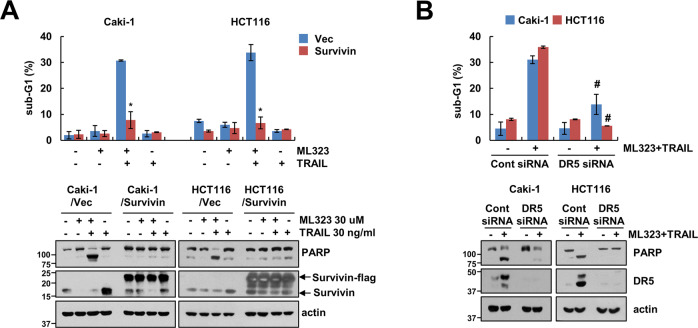
Downregulation of survivin and upregulation of DR5 contribute to ML323-mediated TRAIL sensitization. Caki-1 and HCT116 cells were transfected with vector and flag-survivin (**A**) or control siRNA or DR5 siRNA (**B**) followed by 30 μM ML323, 50 ng/mL TRAIL, or combination for 24 h. The sub-G1 population and protein expression were determined using flow cytometry and western blotting, respectively (**A, B**). Values in the graphs (**A**, **B**) represent the mean ± SD of three independent experiments. *P < 0.01 compared to combination of ML323 and TRAIL in vector. #*P* < 0.01 compared to combination of ML323 and TRAIL-treated control siRNA-transfected group.

Our results showed survivin downregulation and DR5 upregulation with ML323 treatment (Fig. 1C). To verify the critical role of these proteins in ML323-mediated TRAIL sensitization, we used overexpression or knockdown systems. Ectopic expression of survivin markedly prevented the apoptosis and PARP cleavage by combined treatment in cancer cells (Fig. 6A and Supplementary Fig. S3A). Moreover, knockdown of DR5 by siRNA inhibited apoptosis in ML323 plus TRAIL-treated cells (Fig. 6B and Supplementary Fig. S3B). These data demonstrated that survivin and DR5 contribute to ML323-induced TRAIL sensitization.

**Fig. 7:**
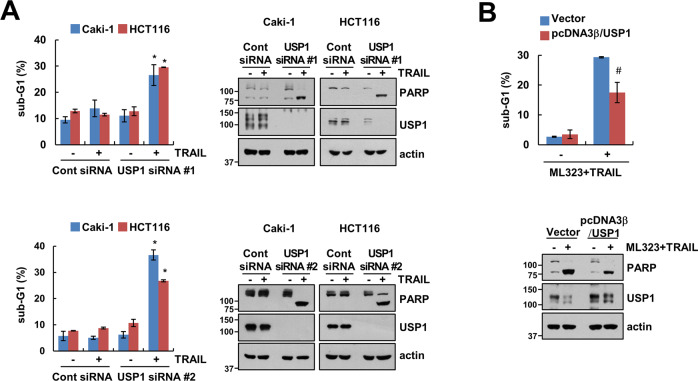
Depletion of USP1 contributes TRAIL sensitization. **A** Caki-1 and HCT116 cells were transfected with control siRNA or USP1 siRNA followed by 50 ng/mL TRAIL for 24 h. **B** Caki-1 cells were transfected with vector or pcDNA3β-USP1 followed by combination of 30 μM ML323 and 50 ng/mL TRAIL for 24 h. The sub-G1 population and protein expression were determined using flow cytometry and western blotting, respectively (**A**, **B**). Values in the graphs (**A**, **B**) represent the mean ± SD of three independent experiments. *P < 0.01 compared to TRAIL-treated control siRNA-transfected group. #*P* < 0.01 compared to combinations of ML323 and TRAIL-treated pcDNA3β-USP1-transfected group.

The role of USP1 in TRAIL-induced apoptosis using specific siRNAs for USP1 was investigated. As shown in Fig. 7A and Supplementary Fig. S4A, TRAIL alone significantly increased apoptosis in USP1 siRNA-treated cells. In addition, ectopic expression of USP1 attenuated the co-treatment with ML323 and TRAIL-induced apoptosis (Fig. 7B and Supplementary Fig. S4B). Therefore, these results suggested that the inhibition of USP1-mediated survivin downregulation and DR5 upregulation can modulate the TRAIL sensitization.

### Anti-tumor effect of ML323 and TRAIL combined treatment in vivo

**Fig. 8:**
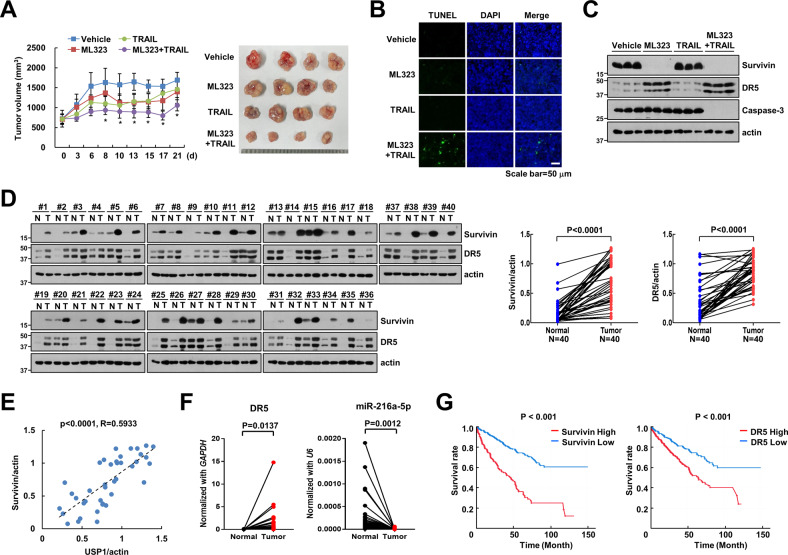
Anti-tumor effect of combination of ML323 and TRAIL in xenograft model. **A**–**C** Mice bearing HCT116 cells were treated with 10 mg/kg ML323, 3 mg/kg GST-TRAIL, combinations, or vehicle (i.p.) for 21 days. Tumor volume and size were measured (**A**). Terminal deoxynucleotidyl transferase (TdT) dUTP Nick-End Labeling (TUNEL) assays were performed to check apoptosis in vivo. scale bar: 50 μm (**B**). Protein expression was measured by western blot analysis (**C**). **D** Examination of survivin and DR5 protein expression in 40 paired primary RCC tissues and corresponding normal adjacent tissues. **E** Correlation analysis of protein expression of USP1/survivin. **F** Examination of DR5 (**left panel**) and miR-216a-5p (**right panel**) mRNA expression in 40 paired primary renal cancer cell (RCC) tissues and corresponding normal adjacent tissues. **G** Analysis of prognostic significance of survivin and DR5 in RCC patients based on TCGA. *P < 0.01 compared to the vehicle. i.p. intraperitoneal injection.

We investigated the antitumor effect of combined treatment with ML323 and TRAIL in vivo to prove the outcomes of in vitro experiments. The co-treatment decreased the tumor volume and increased the apoptotic signal in the xenograft model (Fig. 8A, B). Moreover, ML323 also downregulated survivin and upregulated DR5 in the tumor tissues of the xenograft model (Fig. 8C). In addition, we performed immunoblotting to survey the expression of survivin and DR5 in non-tumor versus tumor tissues. Most tumor tissues exhibited markedly higher expression levels of survivin and DR5 than the non-tumor tissues (Fig. 8D). Thus, the tumor tissue showed a positive correlation between USP1 and survivin protein levels (Fig. 8E). The mRNA of miR-216a-5p in tumor tissues was significantly lower than that in non-tumor normal tissues, whereas DR5 mRNA expression was higher in renal tumor tissues (Fig. 8F). As shown using Kaplan–Meier plotter analysis, the expression of survivin and DR5 was inversely correlated with the progression-free survival of patients with renal carcinoma (Fig. 8G). These data demonstrated that USP1 positively correlates with survivin, while miR-216a-5p is negatively correlates with DR5 mRNA expression.

## Discussion

Our results demonstrated the novel function of USP1 as a DUB to regulate survivin stability. USP1 also functions as a repressor of DR5, which is mediated by miR-216a-5p. USP1 inhibition downregulated miR-216a-5p expression and sequentially upregulated DR5 expression. Combined treatment with a USP1 inhibitor, ML323, and TRAIL-induced apoptosis via survivin downregulation and DR5 upregulation in cancer cells but not in normal cells. These results suggested that ML323 can be an attractive TRAIL sensitizer.

USP1 has been identified as a major regulator in the repair process of DNA damage by stabilizing FANCD2 and PCNA [10, 11]. Many studies have reported the involvement and function of USP1 in cancer metastasis and proliferation. For example, USP1 increases the expression of pro-metastatic genes, especially nuclear protein KPNA2, and USP1 deubiquitinates KPAN2 by interacting with KPAN2, thereby promoting the metastasis of breast cancer via stabilization of KPNA2 [30]. In addition, YAP/TAZ, a key effector of the Hippo signaling pathway, increases protein stability via USP1, resulting in increased proliferation and migration of breast cancer cells [31]. USP1 also induces deubiquitination and stabilization of Snail, metastasis, and resistance to platinum in ovarian cancer [18]. Furthermore, catalytically active USP1-mediated ULK1 stabilization contributes to the regulation of autophagy in cancer, and canonical autophagic flux is impaired in USP1-depleted cells through degradation of ULK1, followed by the inhibition of osteosarcoma cell growth [32]. Although USP1 is a regulator of diverse substrate and cellular biological processes, USP1-targeted substrates and the underlying mechanisms of cancer cell death remain unclear.

In our study, to identify new substrates of USP1 in cancer cell death, we investigated alterations in the major regulators of apoptosis using genetic (siRNA) and pharmacological inhibitors. First, we found that USP1 inhibition downregulated survivin protein levels (Fig. 1C–E). In addition, USP1 directly bound to survivin and suppressed its ubiquitination through its catalytic activity (Fig. 2F, G), and the overexpression of survivin significantly inhibited apoptosis induced by the co-treatment with ML323 and TRAIL (Fig. 6A). In RCC, USP1 and survivin were highly expressed in tumor tissues compared to normal tissues, thereby indicating a positive correlation and poor prognosis (Figs. 1A, B and 8E, G). Therefore, we discovered that survivin is a specific substrate of USP1 and is associated with the inhibition of USP1-mediated sensitization of cancer cells to anti-cancer drugs.

Second, ML323 also upregulated DR5 mRNA and protein expression (Figs. 1C, 3B), and we found an increase in DR5 by ML323 at the post-transcriptional level. Because USP1 modulates the ubiquitination and degradation of target substrates through post-translational regulation, we postulated that USP1 indirectly affects DR5 upregulation. miRNAs, which are small noncoding RNA, inhibit mRNA stabilization and translation by binding to the 3′-UTR region of mRNA [33]. Ovcharenko et al. reported that the overexpression of miR-216 reduced caspase activation by interacting with DRs and prevented TRAIL-mediated apoptosis in breast cancer [34]. To support the relation between miR-216a-5p and ML323-induced DR5 mRNA upregulation, we used a specific miR-216a-5p mimic and found that ML323-induced DR5 upregulation was suppressed by transfection of miR-216a-5p mimic (Fig. 4C, D). In addition, RCC tumor tissues showed DR5 mRNA upregulation and miR-216a-5p mRNA downregulation, indicating an inverse correlation. These data suggested that ML323 increases DR5 mRNA expression via the repression of miR-216a-5p. Moreover, several miRNAs are involved in the TRAIL-induced apoptotic pathway [35]. In particular, miR-221 and -21 indicate the tolerance, whereas miR-7 increases the sensitivity to TRAIL [34, 36, 37]. However, the expression of these miRNAs was not altered by ML323 treatment, and the overexpression of these miRNAs using each mimic did not inhibit ML323-induced DR5 upregulation (Fig. 4A, B). Thus, we ruled out the involvement of three miRNAs (miR-221, miR-21, and miR-7) in ML323-mediated DR5 upregulation and TRAIL sensitization. We identified eight putative transcription factor binding sites (E2F7, TGFB1, PRDM1, TAZ, RNF2, KDM6B, TLE3, and AP1) in the miR-216-5p promoter region through TransmiR v2.0 programs [38]. Mussell et al. reported that knockdown of USP1 decreases TAZ protein level using DUB siRNA library, and USP1 interacts and deubiquitinates TAZ, resulted in the increase of cancer cell proliferation [31]. Therefore, we proposed the possibility that the TAZ transcription factor may be involved in USP1-mediated regulation of miR-216-5p. We required to further studies to investigate the molecular mechanisms of USP1/TAZ/miR-216-5p axis.

**Fig. 9:**
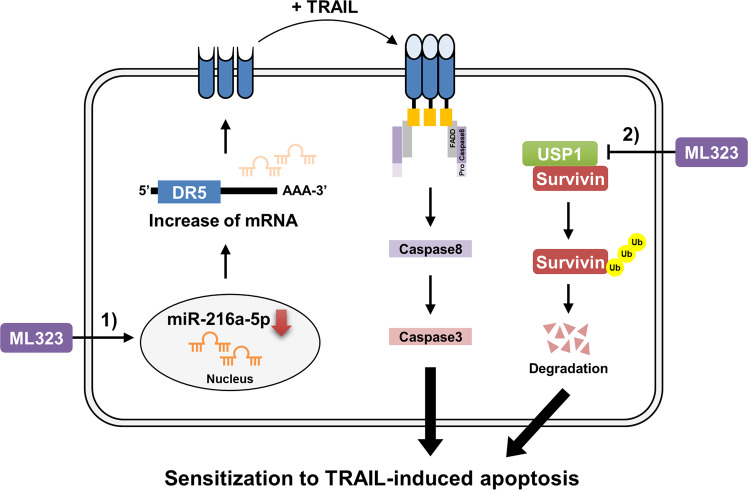
Schematic representation of the mechanism of sensitization to anticancer drugs by USP1 depletion. Two signaling pathways are involved in ML323-mediated TRAIL sensitization. (1) ML323 decreases miR-216a-5p expression at the transcription level, stabilizes DR5 mRNA, and increases DR5 expression on cancer cell surface. (2) USP1, highly expressed in renal cancer, interacts with survivin and induces deubiquitination of survivin. When USP1 is inhibited by ML323, survivin is ubiquitinated and degraded. Eventually, sensitivity of TRAIL is increased by upregulation of DR5 and downregulation of survivin in ML323 treatment.

USP1 is a new DUB capable of stabilizing survivin, and USP1 depletion enhances TRAIL-mediated cancer cell death by degrading survivin. Moreover, other pharmacological USP1 inhibitors induce DR5 mRNA expression through downregulation of miR-216a-5p, eventually increasing sensitivity to TRAIL in cancer (Fig. 9).

## Supplementary information

Supplementary Figure 1

Supplementary Figure 2

Supplementary Figure 3

Supplementary Figure 4

Supplementary information

uncropped western blots

checklist

## Data Availability

The datasets used and analyzed in this study are available from the corresponding author.
