# Supplementary figures and images for: Inhibition of USP1 enhances anticancer drugs-induced cancer cell death through downregulation of survivin and miR-216a-5p-mediated upregulation of DR5
Source: Cell Death Dis. 2022 Sep 24;13(9):821. doi: 10.1038/s41419-022-05271-0 (PMC9509337; doi:10.1038/s41419-022-05271-0)

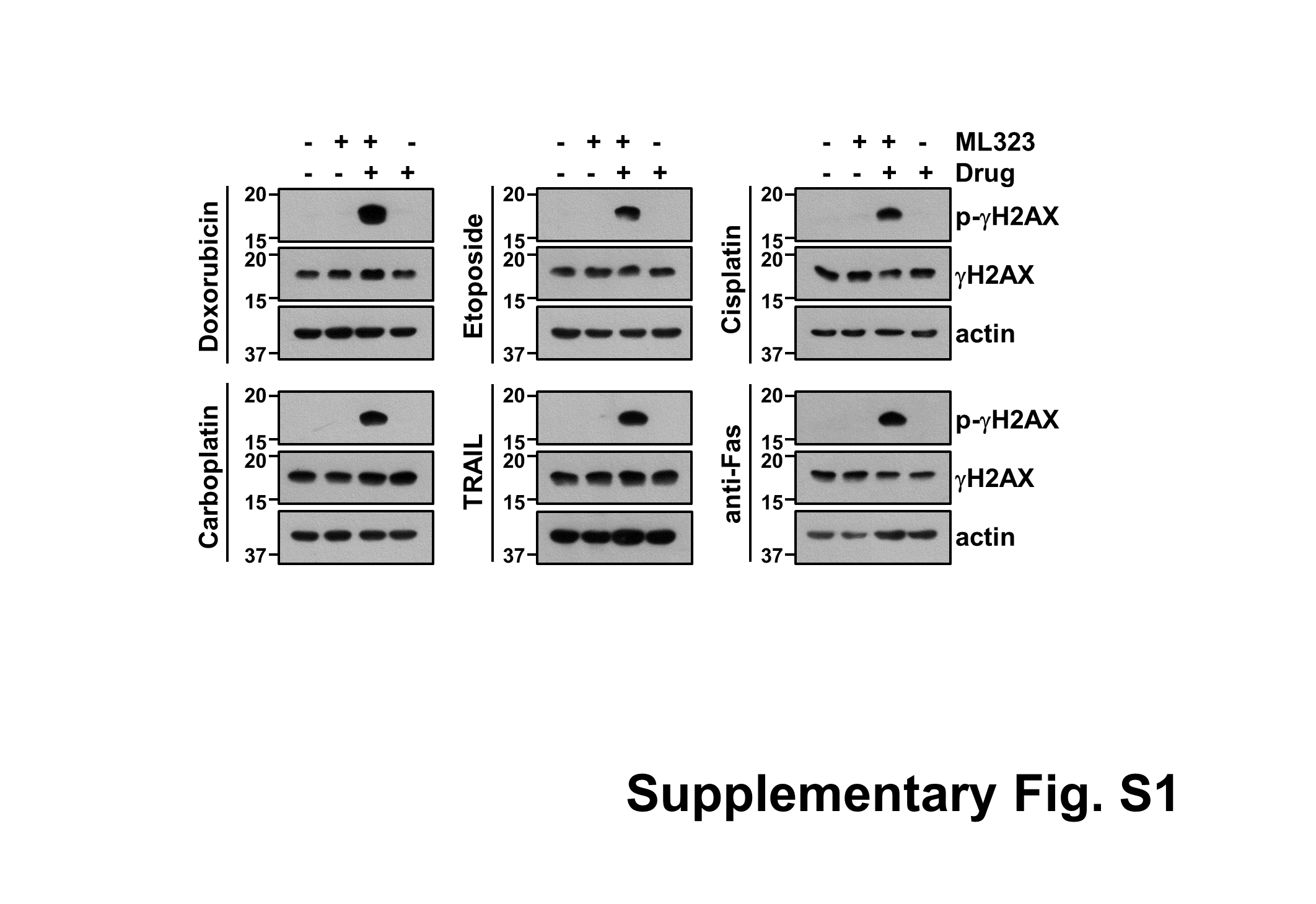

Supplement: Supplementary file 1 — Supplementary Figure 1 [file 41419_2022_5271_MOESM1_ESM.tif]

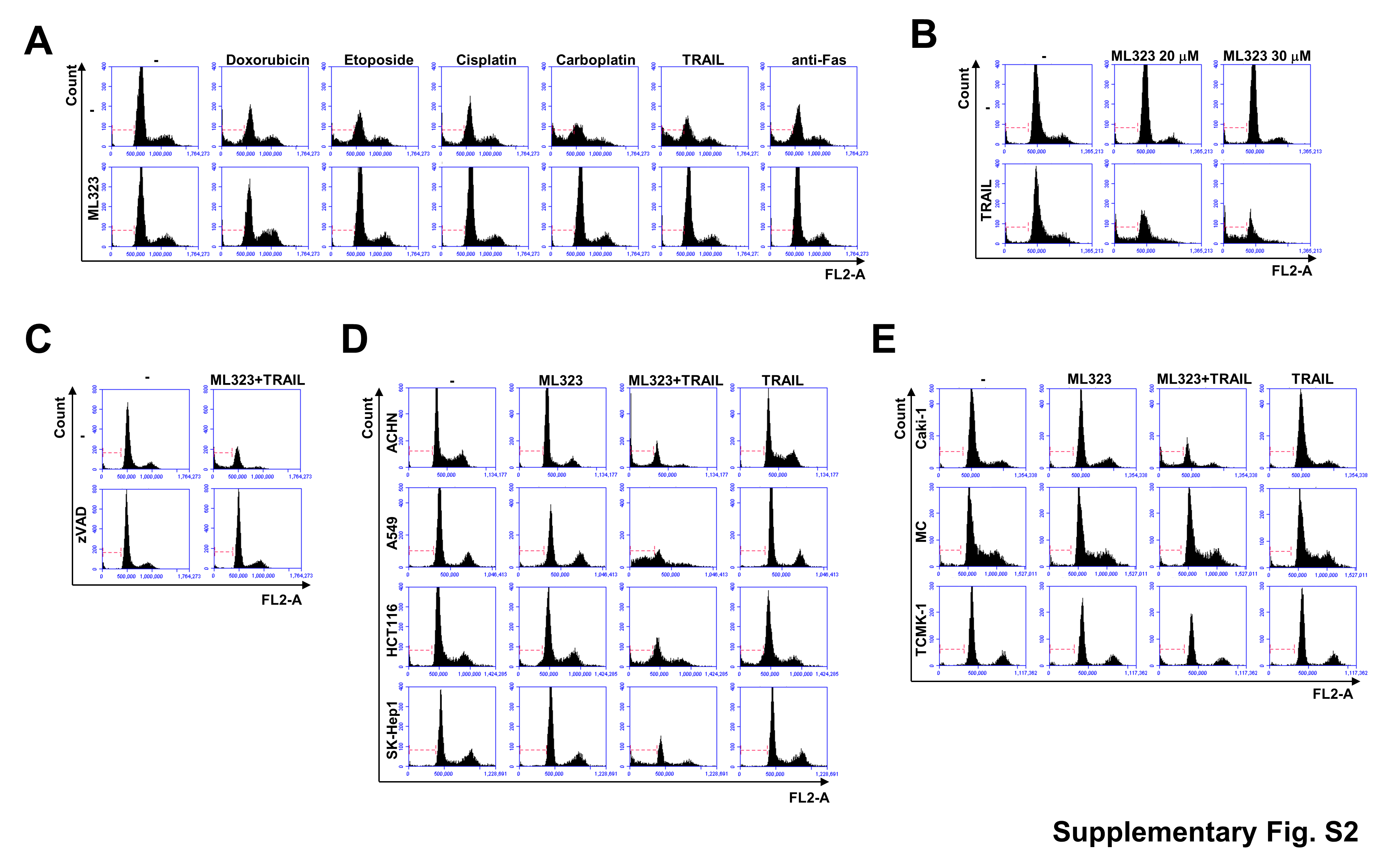

Supplement: Supplementary file 2 — Supplementary Figure 2 [file 41419_2022_5271_MOESM2_ESM.tif]

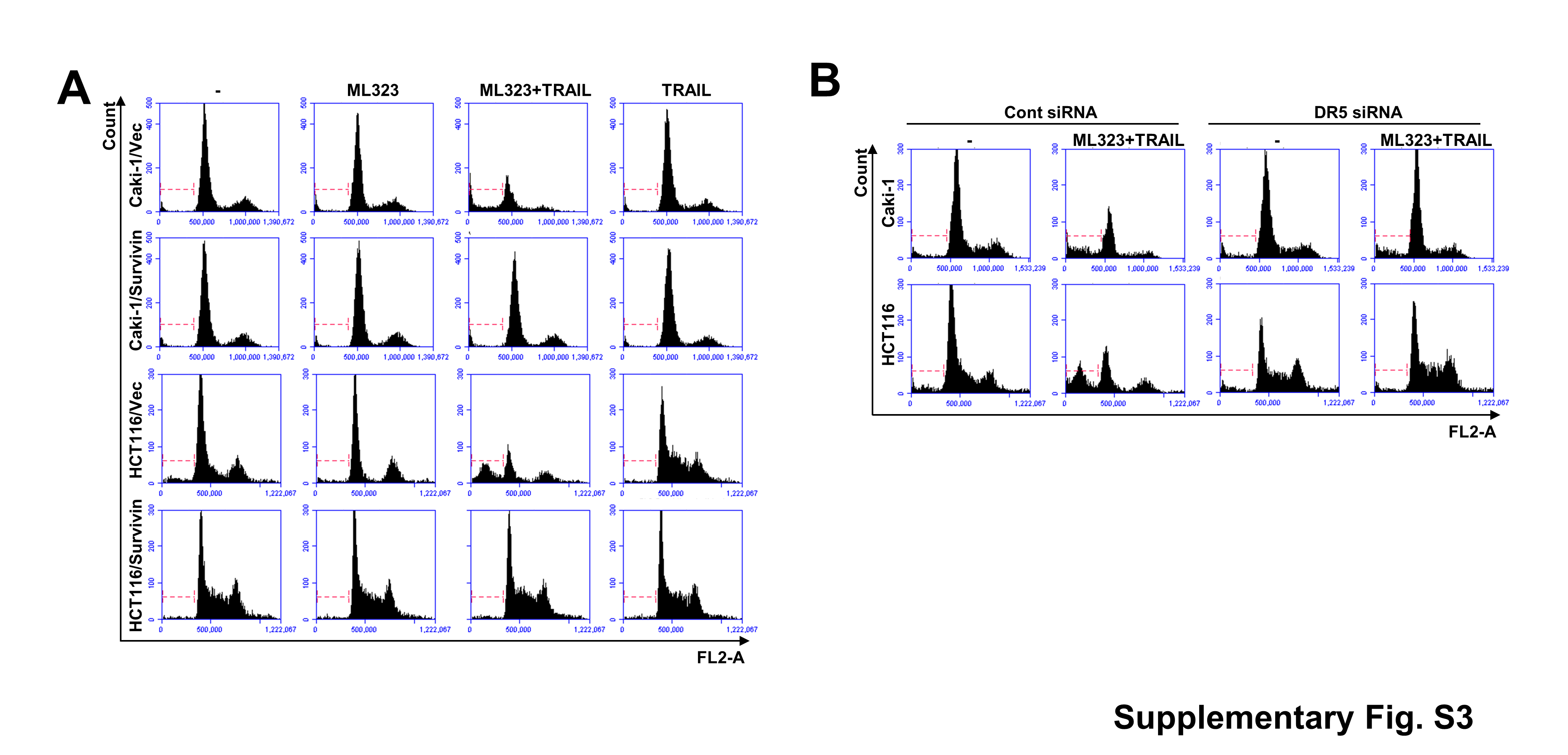

Supplement: Supplementary file 3 — Supplementary Figure 3 [file 41419_2022_5271_MOESM3_ESM.tif]

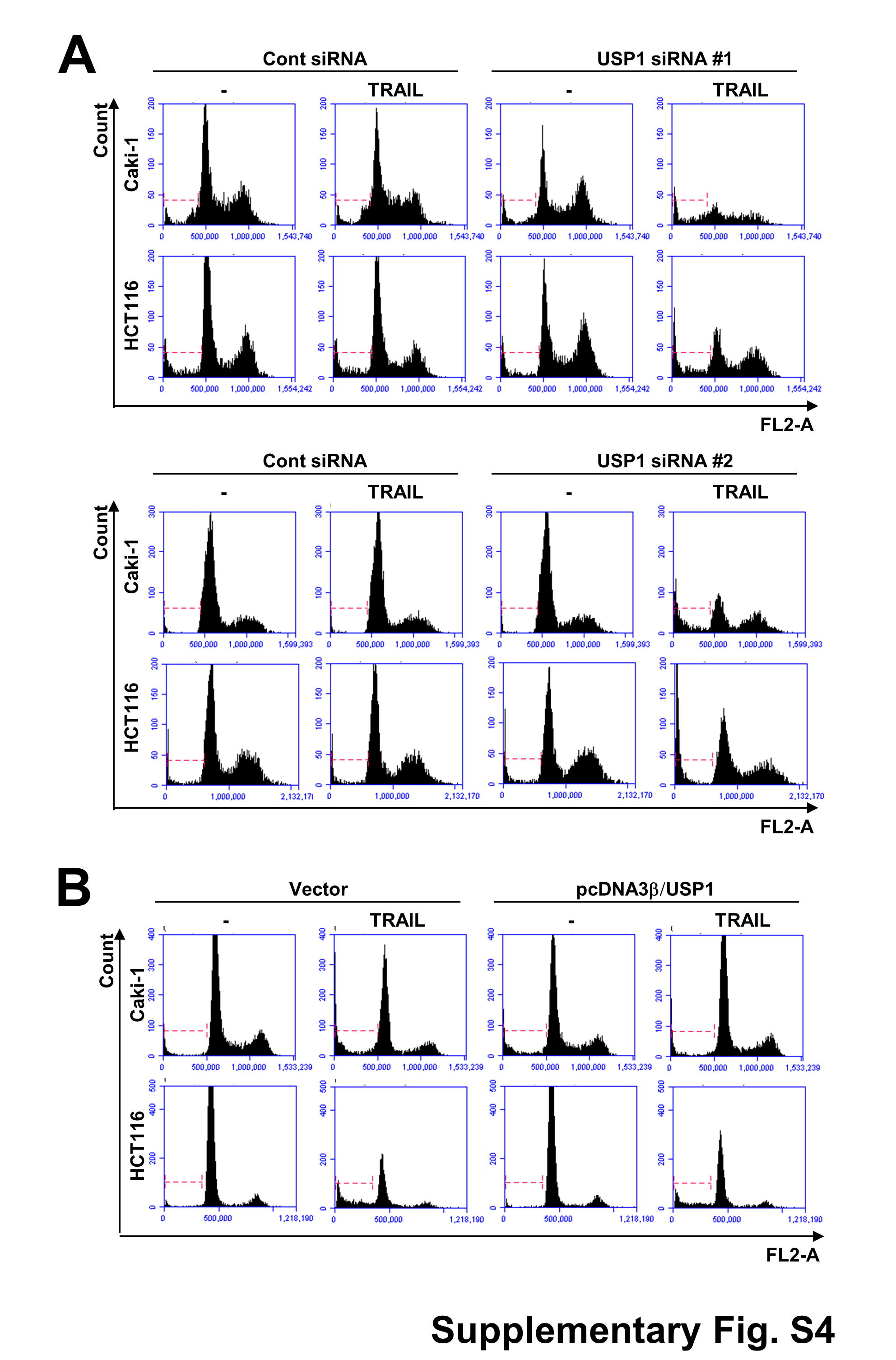

Supplement: Supplementary file 4 — Supplementary Figure 4 [file 41419_2022_5271_MOESM4_ESM.tif]
