# Supplementary material for: Inhibition of USP1 enhances anticancer drugs-induced cancer cell death through downregulation of survivin and miR-216a-5p-mediated upregulation of DR5
Source: Cell Death Dis. 2022 Sep 24;13(9):821. doi: 10.1038/s41419-022-05271-0 (PMC9509337; doi:10.1038/s41419-022-05271-0)
Supplement: Supplementary file 5 — Supplementary information [file 41419_2022_5271_MOESM5_ESM.docx]

**Supplementary Information**

**Supplementary Figure legends**

**Supplementary Tables S1**

**Supplementary Methods**

**Fig. S1 Combined treatment of ML323 and anticancer-drugs increases DNA damage.** Caki-1 cells were treated with a combination of 1 μM doxorubicin, 3 μg/mL etoposide, 30 μM cisplatin, 200 nM carboplatin, 50 ng/ml TRAIL, and 500 ng/mL anti-Fas in the presence or absence of 30 μM ML323 for 24 h. The protein expression was determined using western blotting.

**Fig. S2 Flow cytometry histogram corresponding to Figure 5. A** Caki-1 cells were treated with a combination of 1 μM doxorubicin, 3 μg/mL etoposide, 30 μM cisplatin, 200 nM carboplatin, 50 ng/ml TRAIL, and 500 ng/mL anti-Fas in the presence or absence of 30 μM ML323 for 24 h. **B** Caki-1 cells were treated with 30 μM ML323, 50 ng/mL TRAIL or combination for 24 h. **C** Caki-1 cells were pretreated with 20 μM zVAD for 30 min and then treated with combination of 30 μM ML323 and 50 ng/mL TRAIL for 24 h. **D, E** Cancer (**D**) or normal cell (**E**) lines were treated with 30 μM ML323, 50 ng/mL TRAIL or combination for 24 h.

**Fig. S3 Flow cytometry histogram corresponding to Figure 6. A, B** Caki-1 and HCT116 cells were transfected with vector and Flag-survivin (**A**) or control siRNA or DR5 siRNA (**B**) followed by 30 μM ML323, 50 ng/mL TRAIL, or combination for 24 h.

**Fig. S4 Flow cytometry histogram corresponding to Figure 7. A** Caki-1 and HCT116 cells were transfected with control siRNA or USP1 siRNA followed by 50 ng/mL TRAIL for 24 h. **B** Caki-1 cells were transfected with vector or pcDNA3β-USP1 followed by combination of 30 μM ML323 and 50 ng/mL TRAIL for 24 h.

**Table S1. Univariate and multivariate Cox regression analysis of overall survival based on TCGA database.**

| **Characteristics** | **Univariate** | | |  | | **Multivariate** | | | |
| --- | --- | --- | --- | --- | --- | --- | --- | --- | --- |
|  | **HR** | **95% CI** | ***P* value** | |  | | **HR** | **95% CI** | ***P* value** |
| **Age (<60 vs ≥60 years)** | **1.99** | **1.215-3.258** | **0.006** | |  | | **1.532** | **0.926-2.535** | **0.097** |
| **Gender (male vs female)** | **0.936** | **0.672-1.302** | **0.693** | |  | | **1.1** | **0.784-1.543** | **0.583** |
| **Stage (I vs)**  **II**  **III**  **IV** | **1.7**  **2.927**  **6.686** | **0.887-3.261**  **1.880-4.557**  **4.415-10.127** | **0.110**  **<0.001**  **<0.001** | |  | | **2.204**  **2.987**  **7.461** | **1.139-4.266**  **1.914-4.66**  **4.887-11.39** | **0.019**  **<0.001**  **<0.001** |
| **Smoking (no vs yes)** | **0.376** | **0.202-0.699** | **0.002** | |  | | **0.286** | **0.152-0.597** | **<0.001** |

HR: hazard ratio, CI: confidence interval

**Statistical analysis**

Univariate and multivariate Cox regression were performed using Statistical Package for Social Sciences software (SPSS Inc., Chicago, IL, USA). Hazard ratio (HR) and 95% confidence interval (CI) were estimated from Cox proportional hazard models.
