# Supplementary material for: Inhibition of USP1 enhances anticancer drugs-induced cancer cell death through downregulation of survivin and miR-216a-5p-mediated upregulation of DR5
Source: Cell Death Dis. 2022 Sep 24;13(9):821. doi: 10.1038/s41419-022-05271-0 (PMC9509337; doi:10.1038/s41419-022-05271-0)

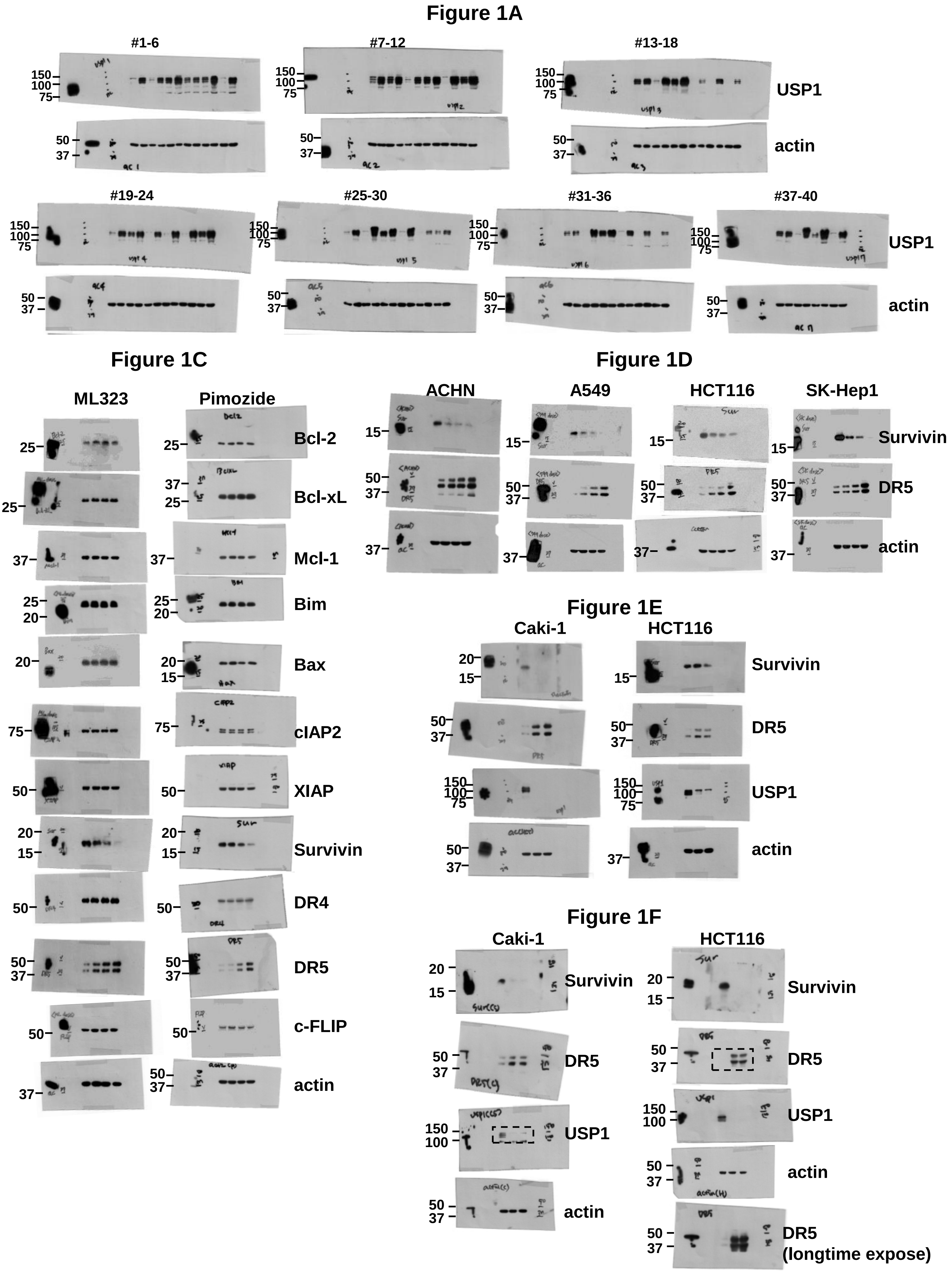
Figure 1A
#1-6
#7-12
#13-18
USP1
actin
#19-24
#25-30
#31-36
#37-40
USP1
actin
150
100
75
50
37
Figure 1C
ML323
Pimozide
Bcl-2
Bcl-xL
Mcl-1
Bim
Bax
cIAP2
XIAP
Survivin
DR4
DR5
c-FLIP
actin
25
20
15
Figure 1D
ACHN
A549
HCT116
SK-Hep1
Survivin
DR5
actin
Figure 1E
Caki-1
HCT116
Survivin
DR5
USP1
actin
Figure 1F
Caki-1
HCT116
Survivin
DR5
USP1
actin
DR5
(longtime expose)

Figure 2B

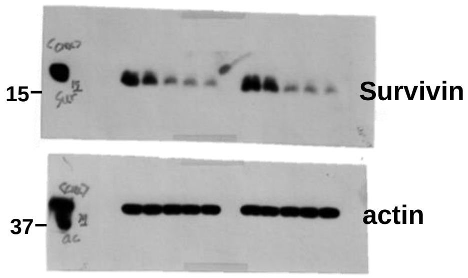

Figure 2C

USP1 siRNA #1

USP1 siRNA #2

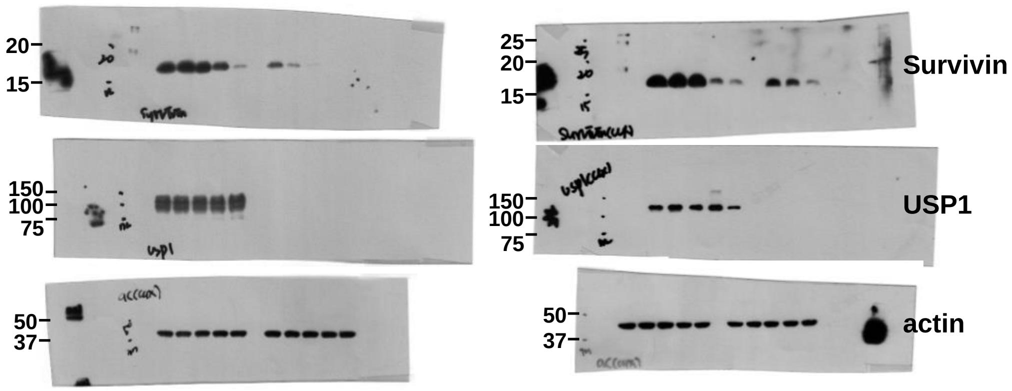

Figure 2D

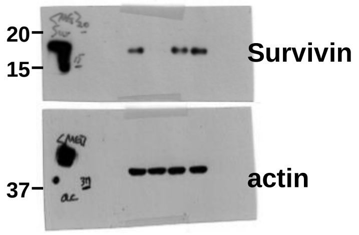

Figure 2E

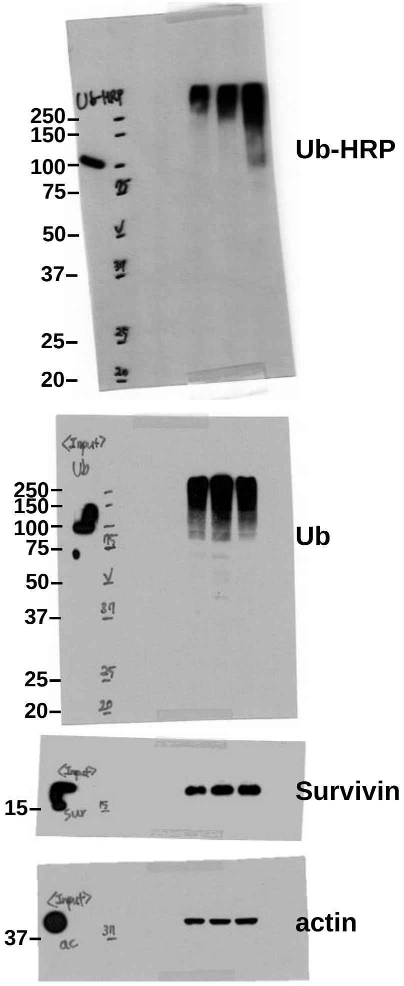

Figure 2F

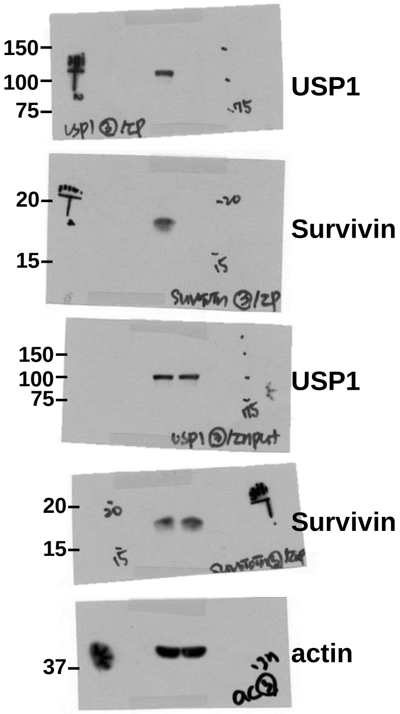

Figure 2G

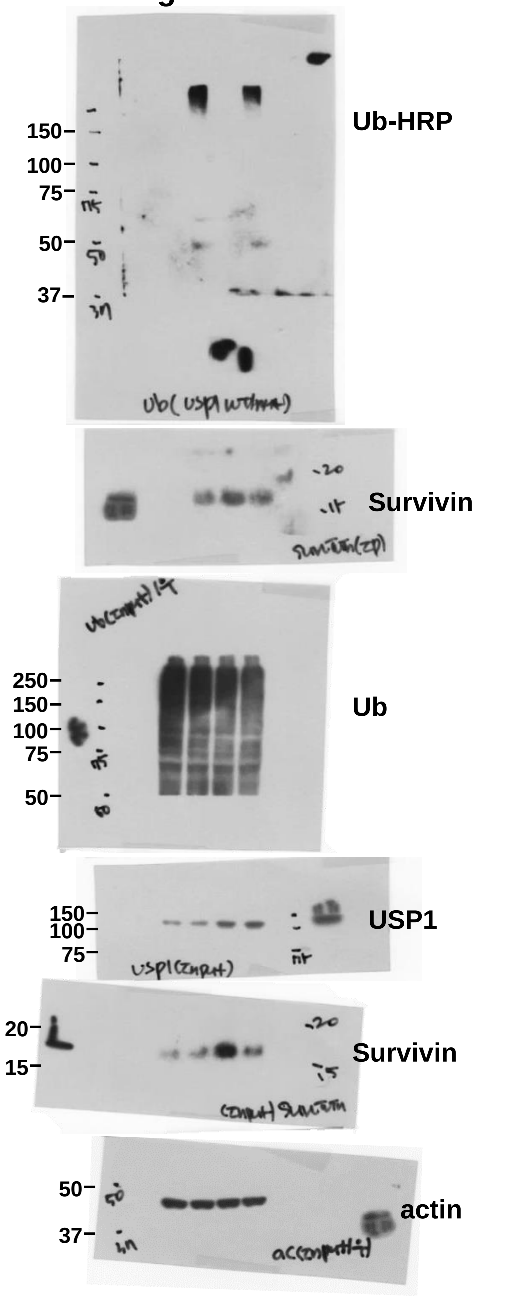

Figure 4B

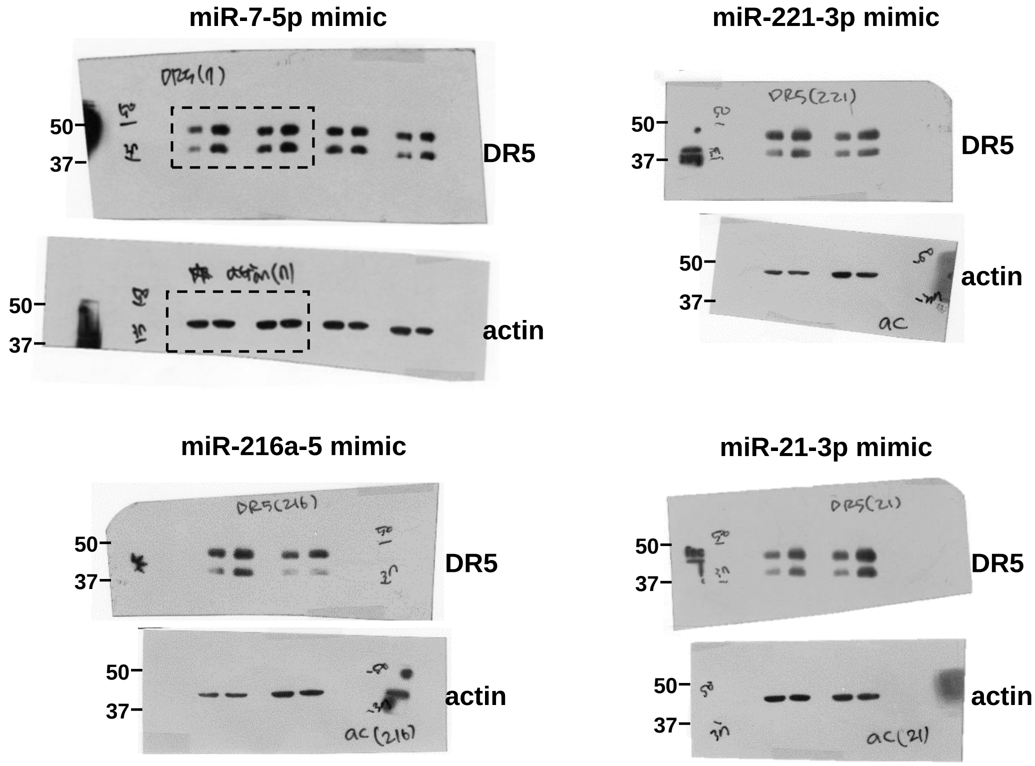

Figure 4C

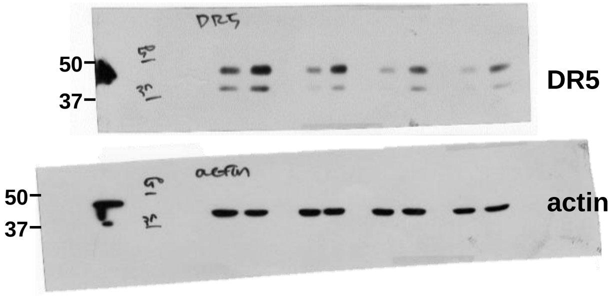

Figure 5B

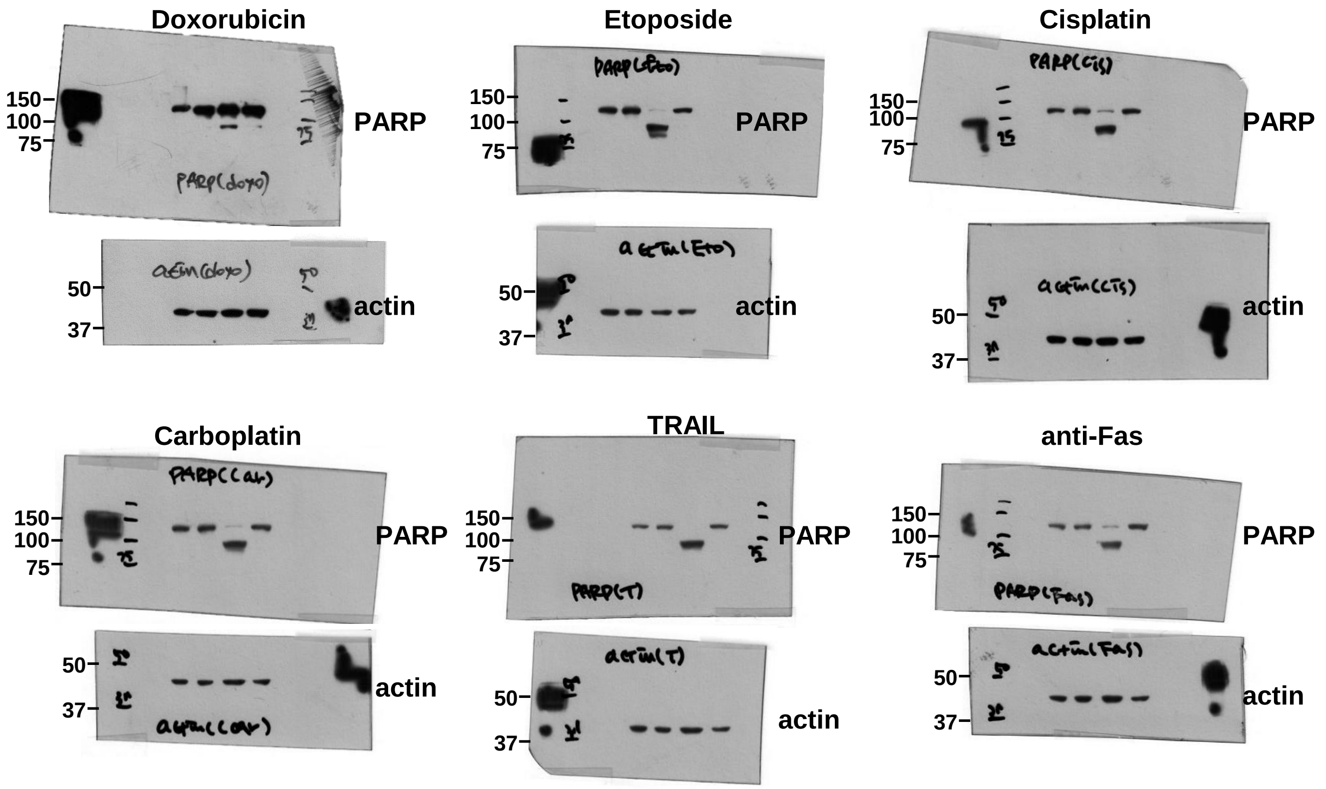

Figure 5C

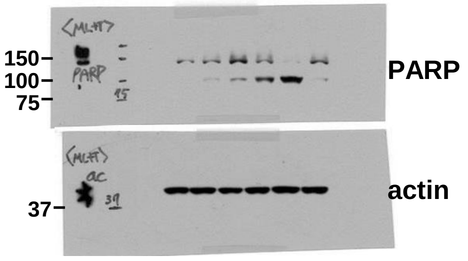

Figure 5G

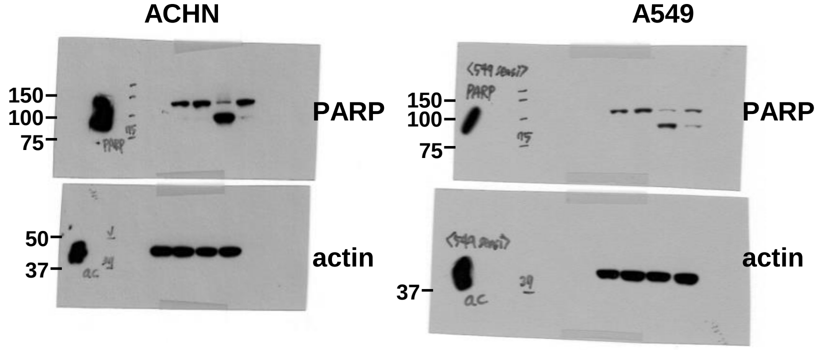

Figure 5F

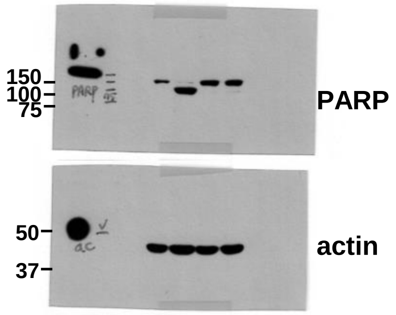

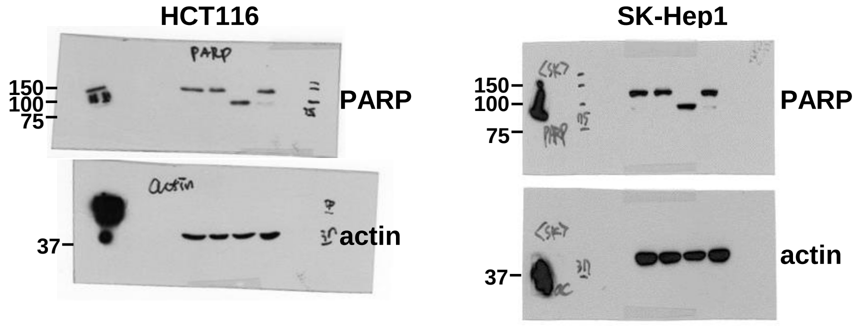

Figure 6A

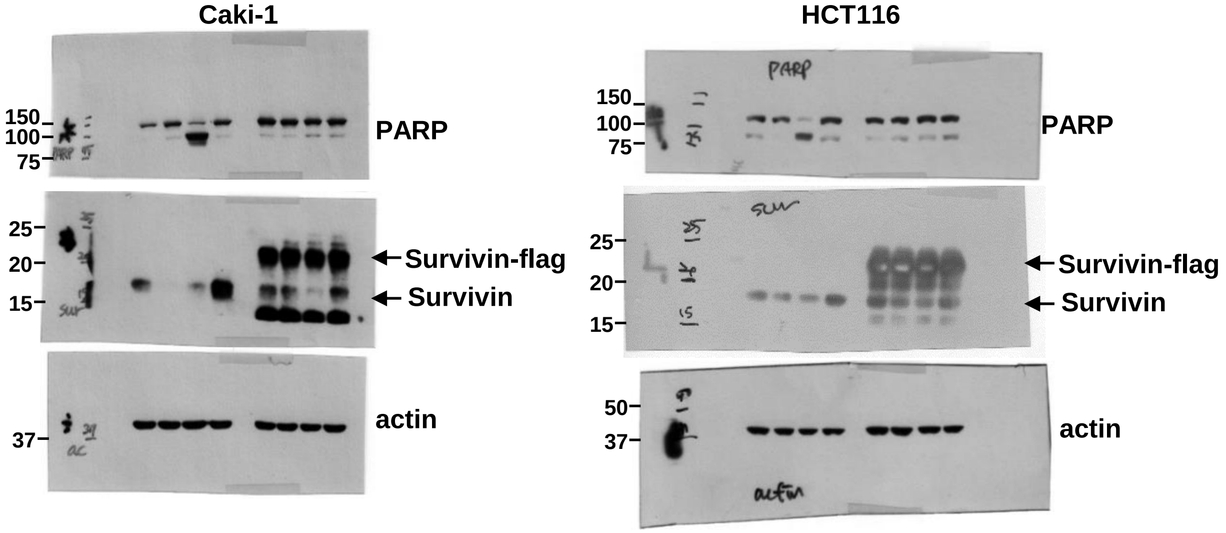

Figure 6B

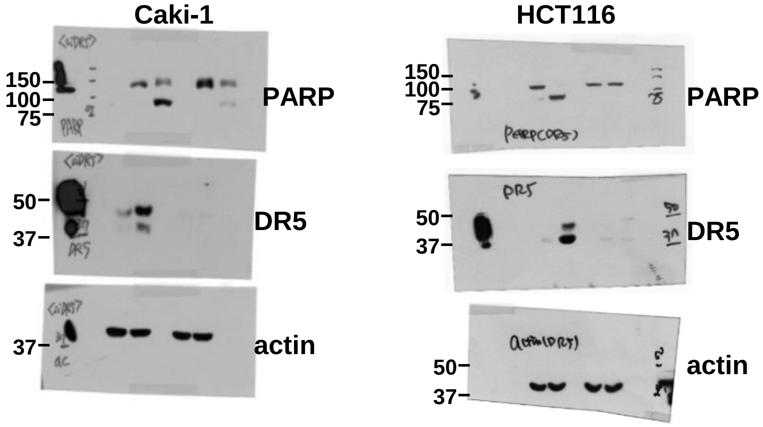

Figure 7A

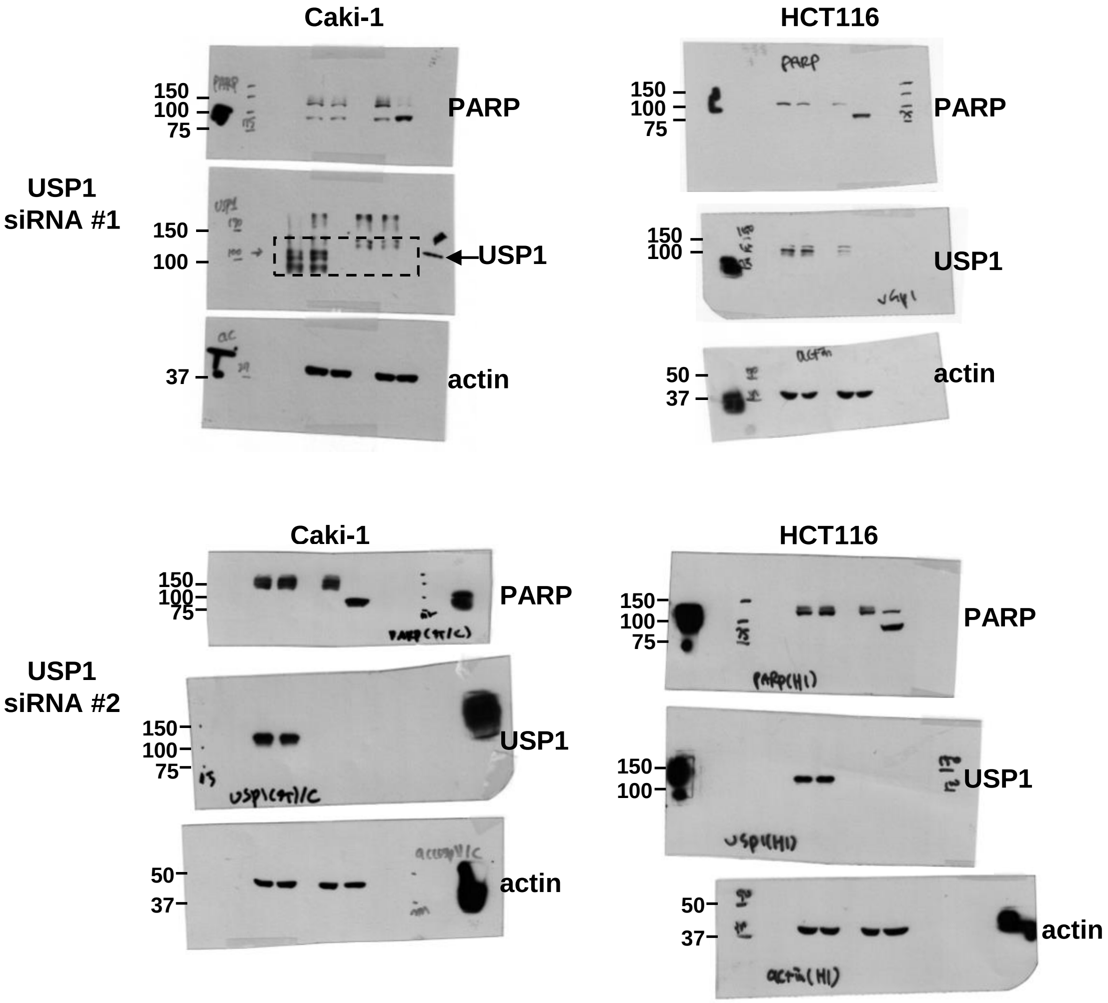

Figure 7B

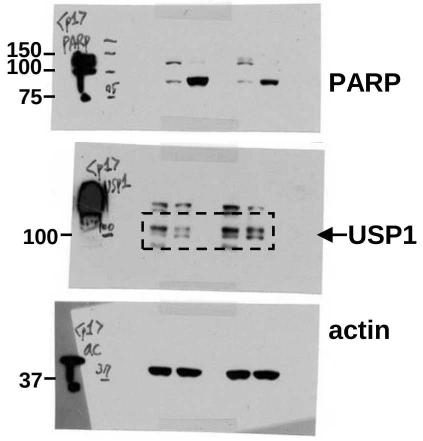

Figure 8A

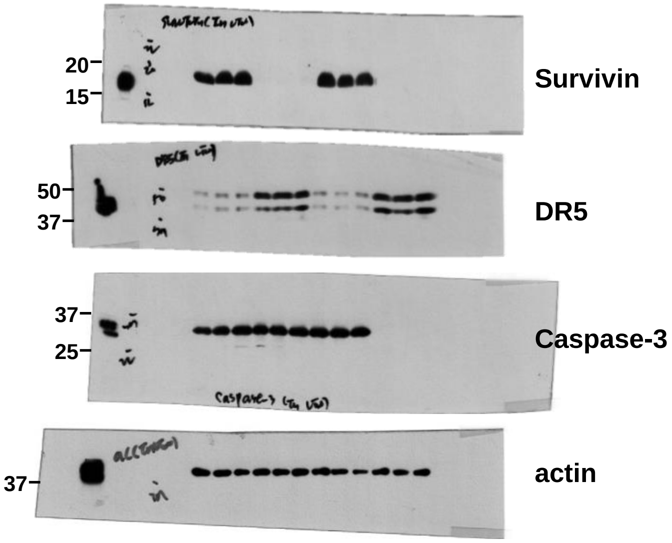

Figure 8D

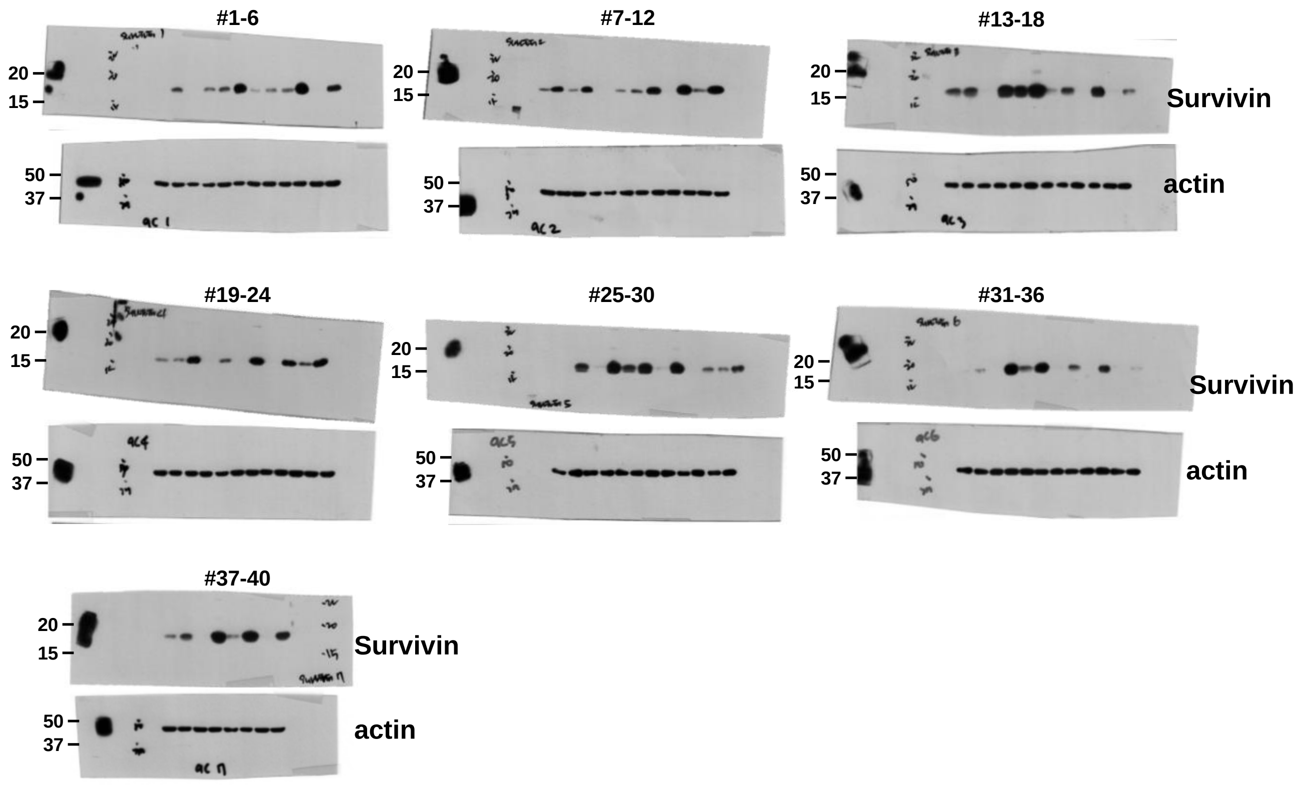

Supplementary Figure S1

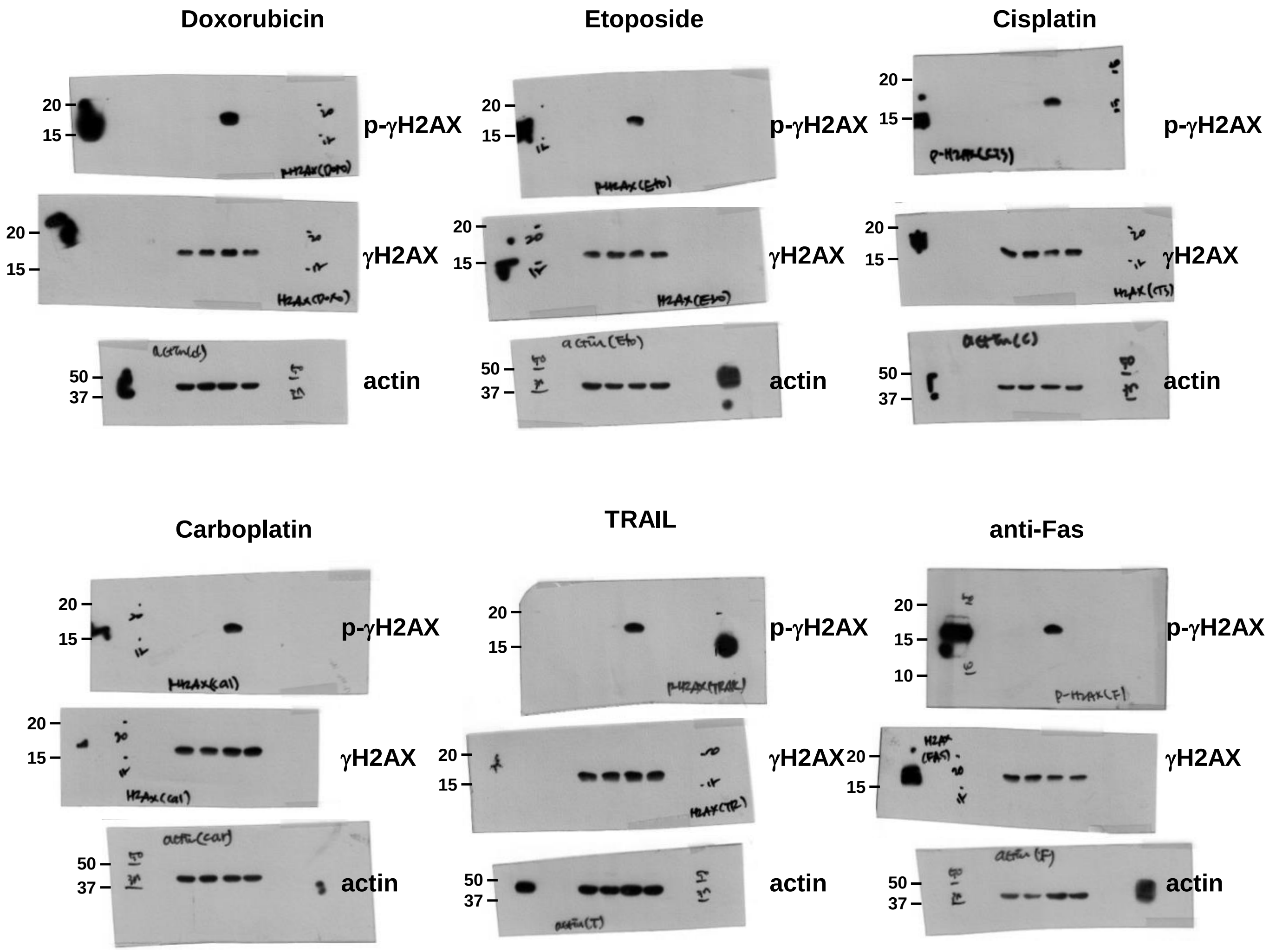

Supplement: Supplementary file 6 — uncropped western blots [file 41419_2022_5271_MOESM6_ESM.pdf]
